# Reflections on the Application of Aboriginal Community Controlled Health Organisations' Research Principles in a Photovoice Food Security Study

**DOI:** 10.1002/hpja.70248

**Published:** 2026-09-24

**Authors:** Emma Louise Chappell, Megan Ferguson, Julie Brimblecombe, Marita Hefler, Caroline Deen, Ellie Chan, Emma Stubbs, Yvonne Cadet‐James

**Affiliations:** ^1^ School of Public Health, The University of Queensland Herston Queensland Australia; ^2^ Menzies School of Health Research, Charles Darwin University Casuarina Northwest Territories Australia; ^3^ Department of Nutrition Dietetics and Food Monash University Notting Hill Victoria Australia; ^4^ College of Medicine & Public Health, Flinders University Brinkin Northwest Territories Australia; ^5^ Apunipima Cape York Health Council Cairns Queensland Australia; ^6^ University Centre for Rural Health, The University of Sydney Lismore New South Wales Australia; ^7^ Poche Centre for Indigenous Health, The University of Sydney New South Wales Australia; ^8^ Central Australian Aboriginal Congress Alice Springs Northwest Territories Australia; ^9^ Indigenous Education and Research Centre, James Cook University Bungalow Queensland Australia

**Keywords:** Aboriginal and Torres Strait islander research, Aboriginal Community Controlled Health Organisation/s (ACCHO/s), ethics, photovoice, research conduct, research principles

## Abstract

**Issue Addressed:**

The conduct of ethical research: the application of ethical practice as guided by Aboriginal Community Controlled Health Organisations' (ACCHOs) research principles.

**Methods:**

A synthesis of two ACCHOs' research principles was conducted, followed by a reflection on the practical application of these principles in a photovoice study in a broader project, the Remote Food Security Project.

**Results:**

The principles synthesis found many commonalities, reflected in six themes with an overall lens of *social justice, fairness, and human rights*. Application of these principles revealed that in general the principles were upheld. Addressing unforeseen challenges was a learning outcome for future research practice. Key factors in application of the principles included Indigenous governance, for example Indigenous representation at all governance levels including the Chief Investigator Group overseeing decision making, Community Advisory Group involvement in implementation design, and participant involvement in the data analysis. Capacity strengthening occurred for all stakeholders including governance groups, research staff and participants, but challenges to engaging and sustaining community researchers were identified with learnings for future research practice.

**Conclusions:**

These two ACCHOs have congruous research principles, reflecting the ethical guidelines on which they are based. In the photovoice study, and within the context of the broader project, there was generally a demonstration of the practical application of these principles, as well as the challenges encountered and valuable learnings.

**So What?:**

Learnings from this study are intended to inform future health promotion research design and ethical health promotion research conduct in partnership with Aboriginal and Torres Strait Islander peoples. Engagement with ethical research principles should be standard practice in research conduct with Aboriginal and Torres Strait Islander peoples, for example through reflecting on ethical guidelines and/or organisational research principles throughout the research process from planning to review and reflection; reporting of this could be too.

## 1 Introduction

Aboriginal and Torres Strait Islander scholarship about ethical research conduct has informed a number of different ethical guidelines [1, 2, 3]. The Lowitja Institute has published a discussion paper covering the history, details and principles of ethical guidelines in health research, which have been informed by Aboriginal and Torres Strait Islander‐specific principles, values and guidelines since 1987 [4]. A central underlying principle of many ethical guidelines is self‐determination, also recognised in the United Nations Declaration of Rights of Indigenous Peoples [5]. There are recent examples in the peer‐reviewed literature of how ethical guidelines have been applied in research practice with Aboriginal and Torres Strait Islander peoples. The Murru Minya project, led by Aboriginal and Torres Strait Islander researchers, aims to examine the practical application of ethical guidelines to research with Aboriginal and Torres Strait Islander peoples, from the perspective of communities, academics and ethics committees, including through consulting with Aboriginal Community Controlled Health Organisations (ACCHOs) [6, 7]. Findings from the project suggest that ethical research practices are lacking, Aboriginal and Torres Strait Islander peoples are less likely than non‐Indigenous peoples to describe research conduct as ethical [8], and research in general does not align with community priorities [9]. Elsewhere, researchers have found that Aboriginal and Torres Strait Islander peoples are not often involved in the conduct and control of research with Aboriginal and Torres Strait Islander peoples, despite ethical guidelines cited as being used [10]. It has been reported that Aboriginal and Torres Strait Islander peoples are more likely to be involved in the conduct and oversight of research conducted by ACCHOs compared with research on which ACCHOs are not leading or partnering [10, 11], however the Murru Minya project also found that ACCHOs are over‐approached to participate in research [9].

**FIGURE 1:**
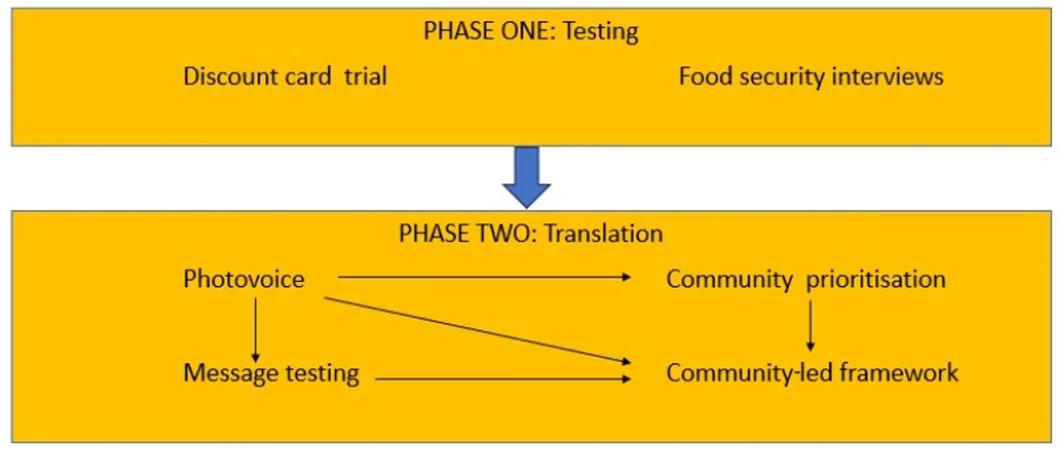
Phases of the remote food security project. Phase one of the broader project included determining the impact of a healthy food price discount strategy on the diet quality of women and children, and conducting interviews on lived experiences of food (in)security in remote communities. Phase two focused on community‐developed solutions, including using photovoice in four communities to develop solutions to improve food security, and then relaying these to representatives from these four communities in community prioritisation workshops, and then to representatives from 10 communities in the development of a community‐led framework, which aimed to develop a knowledge sharing/translation plan for improving food security. Photovoice results also informed the framing study where messages around solutions to improve food security were tested with members of the public, in order to increase the acceptability of proposed policy solutions [14].

ACCHOs, through their delivery of holistic, comprehensive and culturally responsive primary health care services to Aboriginal and Torres Strait Islander peoples [12], are well positioned to guide research conduct [13]. In 2021, two of these ACCHOs, Apunipima Cape York Health Council (Apunipima) and Central Australian Aboriginal Congress (Congress), partnered with the University of Queensland to co‐lead the Remote Food Security Project (Figure 1) (hereafter referred to as ‘the broader project’), with the aim of addressing the high prevalence of food insecurity reported for remote Australia (51%) [15]. Apunipima and Congress provide services to 11 Cape York communities, and nine remote Central Australian communities and the town of Alice Springs respectively [16, 17]. Both ACCHOs have formulated research position statements, strategies and principles relevant to research conduct [16, 18, 19, 20, 21], informed by the work of Aboriginal and Torres Strait Islander scholars, designed for researchers conducting research in partnership with their member communities. These were designed in consultation with communities and with reflection on ethical guidelines. In line with their community‐endorsed food security position statements, the ACCHOs partnered with researchers to address food security [14]. This partnership developed a governance structure with Aboriginal and/or Torres Strait Islander peoples at each decision‐making level. In practice, project governance processes were established, whereby Aboriginal and/or Torres Strait Islander staff members were represented on all decision‐making project groups, and in community, project staff were advised by Community Advisory Groups. The partnership was also represented in agreements (A Multi‐Institutional Agreement (MIA) with Apunipima and a subcontract with Congress). These agreements ensured decisions around project conduct, funding, intellectual property, publications, confidentiality, conflict of interest, publications termination, indemnity and insurance, were outlined and agreed to be all partners including ACCHOs. The photovoice study within these partnerships was conducted with 25 participants in total, over 4 weeks per community (two communities in Cape York [Northern Queensland] and two in Central Australia, Australia) in 2022. It aimed to explore food security and solutions to improve food security in remote Aboriginal and/or Torres Strait Islander communities from the perspective of caregivers of children [22]. The aim of this study herein was to synthesise the research principles of two ACCHOs, and then reflect on how a photovoice study, nested within the broader project, applied these research principles.

During the original co‐design of the broader project, Apunipima identified parents and carers of children under five in remote communities as the target population [18], and determined photovoice to be a suitable method based on Apunipima's experience with photovoice in a community in Cape York. Congress supported this approach. Photovoice has been used in research globally since the 1990s to engage participants to ‘identify, represent and enhance their community.’ [23]. It is a participatory research method, consisting of a series of steps including community problem identification, training, discussion and dissemination [24]. It has origins in empowerment education, feminist theory and documentary photography [24, 25]. The original authors of photovoice stated the method aims to enable people to record and reflect a community's strengths and concerns, to promote critical dialogue through group discussion and to reach policy makers [23].

Prior to the development of this study, the research principles of the ACCHOs informed the development and conduct of the broader project, including the photovoice study, through the contribution of ACCHO team members. The current article is a retrospective reflection on the implementation of the photovoice study, using the framework of ACCHO principles. Throughout the photovoice study, there were formal and informal avenues used for reflection and monitoring in the form of meetings within and between the levels of the governance structure.

The purpose of this current study (hereafter, ‘this study’) was to contribute to the emerging literature on the application of ethical guidelines—and specifically in this case, ACCHO principles based on ethical guidelines—in the conduct of research with Aboriginal and Torres Strait Islander peoples. This was determined by Aboriginal and Torres Strait Islander and other researchers who, as a collective, wanted to reflect on and learn from our research practice in applying the research principles of the two ACCHOs in the study. This study reflects on how research principles informed by Indigenous Knowledges were enacted in practice in a photovoice study. The principles synthesis component of the study was developed by a senior Aboriginal researcher and (at the time of the research) Research Coordinator at Apunipima. Learnings from this study are intended to inform future research design and conduct for research teams and add to the evidence in conducting research in partnership with Aboriginal and Torres Strait Islander peoples. While research principles are widely recognised as crucial for the ethical conduct of such research, there is little evidence of how this has been applied or evaluated.

## 2 Methods

### 2.1 Ethics

Research approval for the broader project was granted by NHMRC. Data for the photovoice study consisted of interview transcripts and photographs. The data for this study are the photovoice protocol, protocol revisions, field notes and two evaluations (described in the methods section). All data are stored in a password‐protected file. Access to all data is restricted and will be retained for up to 5 years after publication and then destroyed in line with the institution policies.

For participants in the photovoice study, participation was optional and they were informed at the first workshop that they could withdraw at any time without being judged or having their health care affected.

The project involved a governance structure that reviewed how the project was being facilitated with Aboriginal and Torres Strait Islander peoples involved at regular intervals and made any adjustments necessary to the project to minimise harm. Application for ethical approval involved consulting a number of ethical guidelines including the National Statement on Ethical Conduct in Human Research, 2007 (Updated 2018) and Ethical conduct in research with Aboriginal and Torres Strait Islander Peoples and communities: Guidelines for researchers and stakeholders. Localised community protocols were adhered to, through consultation with Community Advisory Groups, for example on preferences for meeting settings, information sharing and respect for cultural activities.

Early recognition of potential conflict issues given working in the cultural interface involving stakeholders from diverse worldviews and cultural values and beliefs resulted in scheduled opportunities for open and frank discussions at all levels of the governance structure. Meaningful engagement and reciprocity between stakeholders in the broader project, the photovoice study and this reflective study was achieved through time spent working on and regular review of the concepts, study design, data analysis and manuscript revisions. This is reflected by the data analysis being shared between Aboriginal and non‐Indigenous coauthors. This study has an accompanying CONSIDER statement (Supporting Information S1) which further outlines responsible research conduct.

### 2.2 Researcher Positionality and Roles

This study was a collaboration between Aboriginal (Y.C.J. (Gugu Badhun), C.D. (Kamilaroi) and E.S. (Pitjantjatjara, Yankunytjatjara, Arabana and Adnyamathanha)) and non‐Indigenous authors (E.L.C., J.B., M.H., E.C.). Indigenous research capacity was built through co‐authors E.C. and C.D. being employed on the broader project, with funding provided from the NHMRC grant; Y.C.J. was a Chief Investigator on the broader project, and was at the time employed by Apunipima. Apunipima and Congress staff, both project‐funded and in‐kind, worked on the project and the organisations provided resources and expertise, including education regarding community protocols. The positionality of the senior Aboriginal author, Y.C.J., was key to the conceptualisation and design of this paper, and funding acquisition for the broader project. Y.C.J., with Community Advisory Groups and the ACCHO board, gave approval for the photovoice study to take place at their ACCHOs. Y.C.J. conceptualised the principles synthesis, having developed their own ACCHO's research principles and having awareness of the research principles from the other ACCHO. M.F., J.B., Y.C.J. and M.H. acted in supervisory roles for the photovoice study. The team of coauthors have expertise in public health nutrition, maternal, adolescent and child health and tobacco control, working with Aboriginal and Torres Strait Islander communities, including using participatory research and translational research to inform quality improvement and policy, and were positioned to give input on design and analysis, as detailed in the methods. All authors participated in informal training and ongoing various continuous professional development opportunities including those delivered by Indigenous peoples, which developed capacity to partner with Indigenous stakeholders.

### 2.3 Study Design

A three‐stage approach was used by research team members to reflect on their application of ACCHO research principles in the photovoice study, within the context of the broader project. First, a synthesis of Apunipima and Congress's research principles was conducted to identify commonalities and differences (conducted by E.L.C., C.D., Y.C.J.). Second, documentation of photovoice study processes was reviewed (E.L.C., M.F.) to map practice to the synthesised themes. Third, the documents were reviewed for evidence about where the photovoice study processes could demonstrate further alignment with ACCHO research principles.

### 2.4 ACCHO Research Principles Synthesis

Two key documents which outlined the ACCHO research principles and informed the broader project were used to create a synthesised set of principles: the *Apunipima Research Policy and Strategy (2014)*, and Congress's *A Guide for Health Researchers Working with Aboriginal People in Central Australia (2021)*.

**FIGURE 2:**
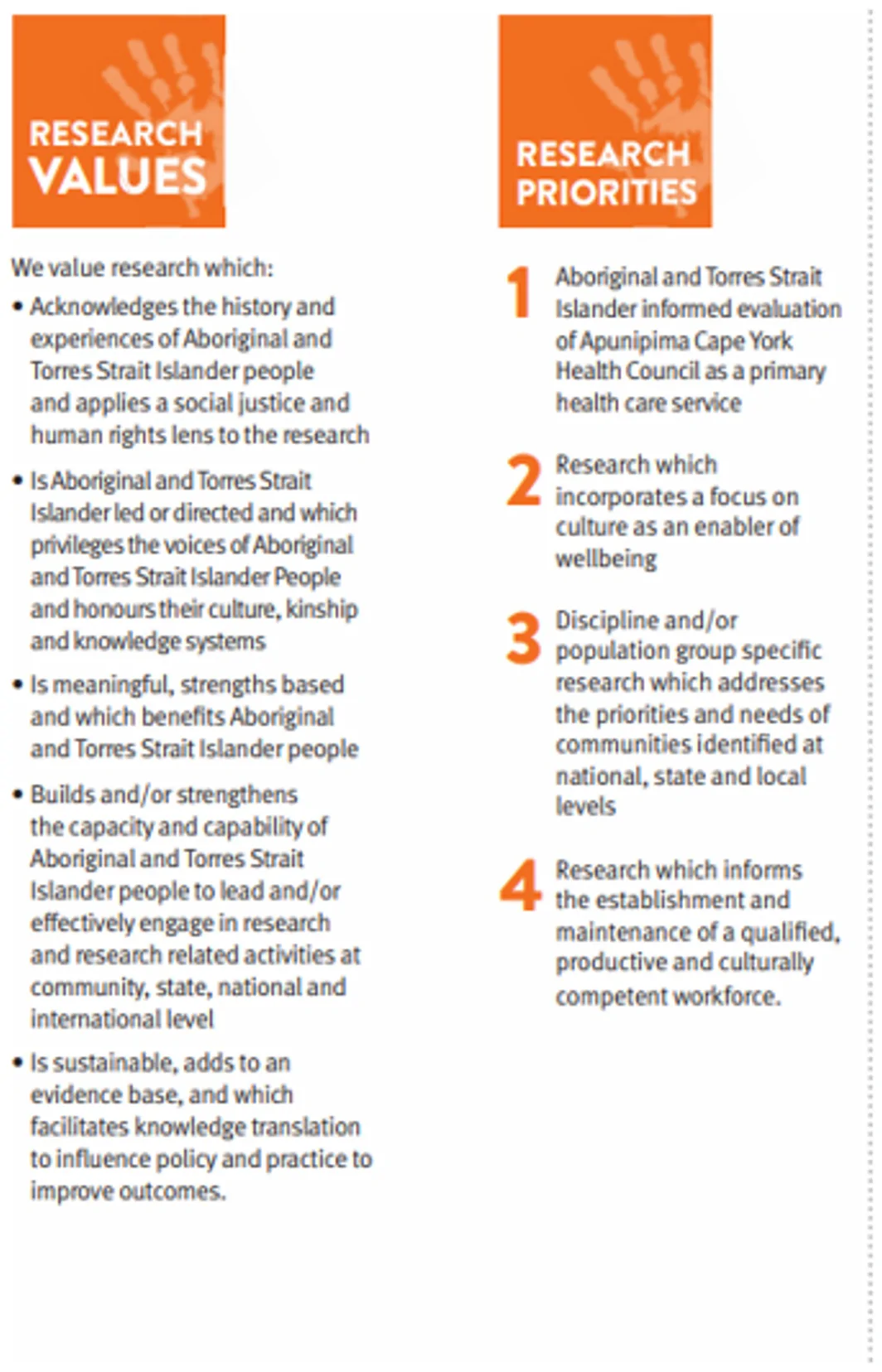
Apunipima research values and priorities.

**FIGURE 3:**
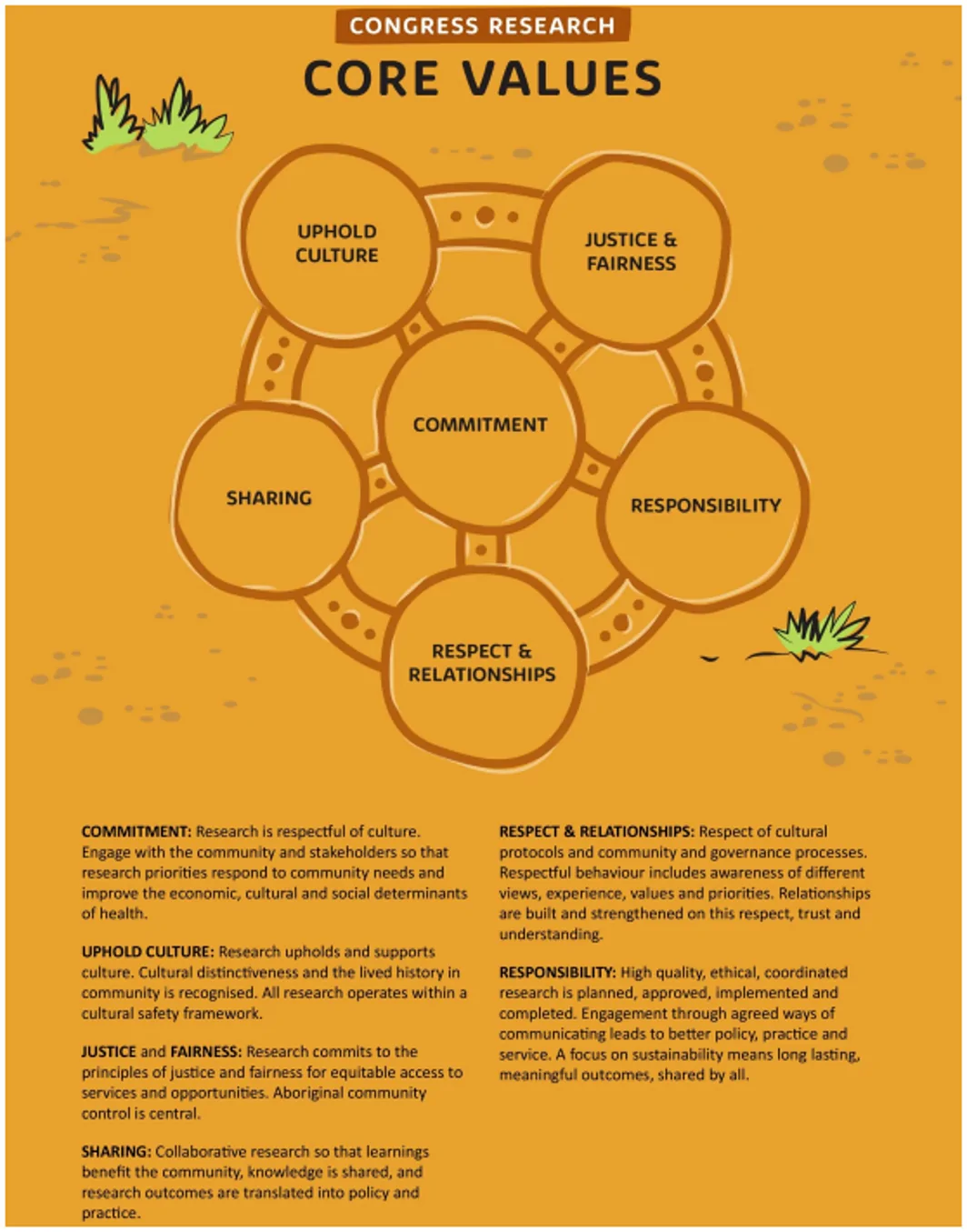
Congress research core values 2019–2023.

After the photovoice study was conducted, the ACCHO research principles (Figures 2 and 3) were carefully reviewed, compared and checked (by E.L.C., M.F. and Y.C.J.). Commonalities and differences between the ACCHO research principles were identified. The theme names were derived deductively, informed by each ACCHO's research principles by comparing existing ACCHO research principle names and selecting or combining text for theme names. Elements of each ACCHOs' research principles were listed side by side, with relevant text bolded. Synthesis notes were developed describing how these research principles were represented across both ACCHOs.

### 2.5 Mapping the Photovoice Study Documents to Synthesised Principles

Documents which detailed the photovoice study processes were reviewed to identify evidence of each ACCHO research principle's application (documents were ‘mapped’ to the principles). The mapping was a novel process designed by the research team. These documents included the outcomes of a process evaluation (a limited evaluation primarily focused on preference for workshop structure), an impact evaluation (conducted with participants in the next steps of the broader project), the photovoice protocol and its adaptations, and field notes by the ACCHO team members (including one Aboriginal team member). An article detailing the co‐design of the broader project [14], the ACCHO food security position statements [18, 20], and an ACCHO research strategy [19] was also included.

The photovoice study documents were reflected on by E.L.C., noting their alignment with the ACCHO research principles. The elements that were planned and worked well with no modification, including planned flexibility within the protocol, were noted. Elements that required modification from the protocol to better align with the ACCHO research principles, and opportunities for better addressing principles were also noted. These were designed to identify opportunities for learning and adaptation inherent in the photovoice study or in the process of reflecting on it. Next, YCJ and ES reflected on initial broad data mapping by EC while reviewing the themes, with YCJ adding more detail to the mapping and the themes. Then E.L.C. and M.F. iteratively reviewed all the photovoice study documents, refined and added more detail to the mapping, validating this with Y.C.J.

## 3 Findings

Here, we first describe the outcome of the research principles synthesis, and then discuss how the photovoice processes aligned with each theme.

### 3.1 ACCHO Research Principles Synthesis

There was congruence between Apunipima and Congress's research principles (Figures 2 and 3). An overarching theme was identified, with six additional themes, to represent a synthesised set of research principles of both ACCHOs (Supporting Information S2). The overarching lens was *social justice, fairness, and human rights*. The six themes were *(i) addressing community‐identified needs and priorities; (ii) Aboriginal and Torres Strait Islander leadership, control and direction; (iii) respect and relationships*; *(iv) a strengths‐based approach which upholds culture; (v) responsible research (benefits and sustainability)*; and *(vi) capacity building/strengthening*.

*Social justice, fairness, and human rights* was identified as an overarching lens as it informed the research approach to this article as well as the broader project and the photovoice study itself. This overarching lens is applicable to all other themes (or principles), as all the themes link to human rights [5, 26]. Further, all the terms in this theme are interconnected. For example, social justice encompasses equity (fairness) and human rights, as well as participation [27], which is addressed through the method of photovoice and the governance structures of the project.

Some ACCHO research principles related to multiple themes, for example Congress's core value of ‘Justice and Fairness’, contributed elements to the overarching lens of *Social justice, fairness, and human rights* and to *(ii) Aboriginal and Torres Strait Islander leadership, control and direction*. Apunipima's call for research to ‘[privilege] the voices of Aboriginal and Torres Strait Islander People[s]’ was found to be related to the overarching lens of *Social justice, fairness, and human rights* and themes *(ii) Aboriginal and Torres Strait Islander leadership, control and direction* and *(iii) respect and relationships*. Ultimately, for the purpose of this synthesis, it was placed with theme *(iii)* for the reason that respect and relationships (depending on the person's capacity and the lens they bring to the research) may encompass privileging Aboriginal and Torres Strait Islander voices.

### 3.2 Mapping of ACCHO Research Principles With Photovoice Study Documents

When mapping the photovoice study documents to the themes (Supporting Information S3), many processes were mapped to multiple themes. For example, respecting cultural protocols through consulting with Community Advisory Groups relates to themes *(i) addressing community‐identified needs and priorities*, *(iii) respect and relationships*, and *(iv) a strengths‐based approach which upholds culture*.

The next sections present each theme with evidence from the photovoice study documents.

### 3.3 Overarching Lens: Social Justice, Fairness and Human Rights

This lens applies to all themes in these findings. The focus of the photovoice study in the context of the family was social justice. The co‐design approach of the broader project had provided the researchers with the opportunity to listen to Aboriginal and Torres Strait Islander voices, with Aboriginal and Torres Strait Islander representation across the governance structures of the project (meeting the human rights element of the theme, including the right of participation) [4]. The photovoice study and the broader project both included activities for knowledge sharing by facilitating workshops where participants were asked to reflect on other participants' photographs, priorities, and solutions (again, the right of participation). These data were then used to identify priorities for community‐led solutions to improve food security (which addressed the fairness element of the theme through developing equitable solutions). Following the photovoice study, the research translation activities provided further avenues for fairness, as community voices were heard throughout the photovoice study and the subsequent community prioritisation workshops, leading to input into local and national solutions to improve food security.

#### 3.3.1 Addressing Community‐Identified Needs and Priorities

The broader study was developed in response to the ACCHOs' identification of food security as a priority through their position statements [18, 20], and Congress's research strategy [19]. The research was made specific to a population because Apunipima identified the target population as women (carers) and young children [18]. Broader project design and funding acquisition for the project involved four Aboriginal Chief Investigators and one Torres Strait Islander Associate Investigator.

The photovoice study supported participants to identify priorities and solutions by the use of a prompt through which participants were asked to discuss their photographs (‘What makes it easy or hard to get or cook food for your family?’) and follow‐up questions. The broader project then facilitated a workshop bringing participants together to further develop priorities and solutions identified through photovoice.

Community needs and preferences were identified through the study's project managers consulting with Community Advisory Groups, which were established for the broader project in each community and comprised Aboriginal people with an interest in health. These Community Advisory Groups informed the research activity, including timing of community visits and appropriate reimbursement for participation as well as being involved in preliminary analysis discussions for the broader project including photovoice. Community members were engaged participants, as evidenced by their continued dedication to finding a way to participate in each workshop (despite time constraints and extenuating circumstances), their engagement in the discussions, and their interest in whether there would be an opportunity to participate in a study like this again in the future.

#### 3.3.2 Aboriginal and Torres Strait Islander Leadership, Control and Direction

The conduct of this research demonstrated Aboriginal and Torres Strait Islander leadership, control, and direction in many ways, with three examples highlighted here.

ACCHO approval: Both ACCHOs have a research approval process that includes assessment of research proposals by majority Aboriginal and/or Torres Strait Islander boards, and then individual community approval. In first establishing the research partnership, the broader project received this approval, including for the photovoice study.

Governance: The established governance structure of the broader project provided Aboriginal and Torres Strait Islander leadership throughout the conduct of the study through the chief investigator group, project working group, the research implementation team (including project managers, Community Advisory Groups and PhD supervisory team), and Aboriginal and Torres Strait Islander control of suggested solutions and priorities.

Project management and data analysis: An Aboriginal Project Manager (CD) led the implementation of the photovoice study in Cape York, including recruitment and co‐leading the planning and conducting of workshops. Aboriginal and Torres Strait Islander researchers and participants were involved in the photovoice data analysis process, with participants checking the themes generated by researchers from the photographs in discussions with one or two participants at a time.

#### 3.3.3 Respect and Relationships

The development of relationships of respect, trust and understanding occurred throughout the photovoice study. Rapport was built through small but important actions, such as providing transport to workshops and printing photographs for participants to keep.

The research protocol was adapted to optimise the relationships built between participants and the project managers, who had frequent contact with participants in earlier stages of the broader project. For example, in response to the first workshop participant directing their answers to the project manager, rather than the PhD student (E.L.C.), managers facilitated discussions thereafter, with E.L.C (or in one community M.F.) taking notes and asking follow‐up questions.

The protocol was also adapted to respect the wishes of participants to share their photovoice stories individually or in a group of two. This was most convenient and/or preferable to them, and demonstrates the flexibility of photovoice as a method.

Participants were offered different options to respect their privacy and/or willingness to share their photographs and accompanying stories. The protocol included de‐identification of photographs and stories, with opportunities to share or not share photographs and stories. There were some examples of participants withdrawing stories or photographs, demonstrating this autonomy.

#### 3.3.4 A Strengths‐Based Approach Which Upholds Culture

The study's theoretical underpinnings of participatory research (and its embedded ACCHO research principles, which are the focus of this article) demonstrate the intent of this research to take a strengths‐based approach. A strength of this study was the involvement of Aboriginal and Torres Strait Islander peoples in all aspects of the study, from initial design through to data analysis and dissemination, ensuring Aboriginal and Torres Strait Islander influence on the research. This included sharing of findings with Apunipima and Congress initially through their research co‐ordinator Y.C.J. and research manager, respectively. Another strength was the partnership with ACCHOs who best know the communities. The photovoice photography prompt was strengths‐based in design (asking about what makes it easier to achieve food security for one's family), and the discussion questions were solutions‐focused and centred on family, community and cultural values (including the use of an Aboriginal‐developed definition of food security which acknowledges food security prior to colonisation by mentioning ‘the food of our ancestors’) [28].

The photovoice process inherently values the sharing of worldviews and experiences by encouraging participants to tell their own stories. Specifically, the protection of Indigenous knowledge and cultural heritage was explicitly stated in the consent process in the statement ‘I understand that the ownership of Aboriginal and Torres Strait Islander knowledge and cultural heritage is retained by the informant and this will be acknowledged in research findings and in the dissemination of the research.’

The research team, with university legal expertise, developed consent and copyright forms for participants to decide whether and how to share their photographs, and secondary consent forms for photographs featuring people.

#### 3.3.5 Responsible Research (Benefits and Sustainability)

The knowledge translation [29] aspects of the photovoice study relate to the research principle of responsible research (benefits and sustainability). The photovoice study results informed the broader project's aims of developing community‐led solutions and informing advocacy efforts. Participants in a workshop for community leaders completed a process evaluation which reported that the photovoice data identified local solutions and had ‘some influence on decision‐making’. Knowledge translation was a multi‐step process within the broader project, that included feeding back to Community Advisory Groups, and sharing results with community representatives in community prioritisation workshops for four communities, and a knowledge exchange workshop that the representatives of 10 communities attended and developed a list of priority solutions to improve food security. These were then used in a submission to the Queensland Government's Gather + Grow Queensland Remote Food Security Strategy (2023–2032) and Action Plan (2023–2025) consultation, and engagement by community members with the National Indigenous Australians Agency's consultation on the National Strategy for Food Security in Remote Aboriginal and Torres Strait Islander Communities, which has informed policy action including around subsidies for essential items in remote stores.

#### 3.3.6 Capacity Building/Strengthening

Research team members' skills in research and communication were developed through formal channels, including working groups and research team meetings, and through informal opportunities, including conversations about the photovoice study (e.g., with remote health centre managers). Research team members also learned from participants—for example, around community strengths of traditional foods, sharing relationships, and participants' worldviews in the context of food security.

In three of the four communities, community researchers worked with project managers to develop plans for identified venues for workshops and informed protocol on camera versus mobile telephone use. They enabled the study to be responsive to participant needs, with the community researchers and Community Advisory Groups becoming valuable informants of the research process.

Whilst there were examples of capacity strengthening, there were also limitations due to various challenges. Working in the context of offers of sporadic paid work opportunities meant there were challenges to further engaging community researchers, who had limited availability to engage in photovoice study implementation (and in one community, in recruitment and planning). There were barriers around identifying suitable people, employment requirements, availability, and limited timeframes. Having greater community researcher involvement could have resulted in a local person co‐leading the workshops and potentially assisting with translation and/or data analysis.

Project managers encouraged participants to use photographs to share their stories by providing cameras and instructions, and replacing lost or faulty cameras. About half the participants took photographs independently, and half requested some research team support, for example in generating ideas for images that represent a story, sourcing props for photographs, or providing transport to photography locations.

All photovoice participants who had contributed photographs and could be contacted were invited to participate in the community prioritisation workshops which followed on from photovoice, further strengthening capacity for involvement in research and knowledge translation. Three photovoice participants participated in these workshops, contributing to discussions about community food security. No participants took up the opportunity to share the collective results themselves at these meetings, instead opting for researchers to share them on their behalf.

## 4 Discussion

There is a growing evidence base of practical examples of the extent to which ethical principles are effectively implemented in practice [6, 7, 8, 10]. We developed and documented our process for our own learnings and also to help address this gap through a novel process involving reflecting on research processes using ACCHO principles (which are informed by ethical guidelines) as a framework.

We found that overall, the photovoice processes established in this study aligned with the ACCHO principles. ACCHO research principles were the driver of the effective and respectful research practices, and the photovoice method, with its participatory principles [23] aligns well with these. The flexible nature of photovoice [24] meant we were able to adapt to participant needs and preferences throughout the research. Through this paper, research capacity at each ACCHO will be further developed through sharing results and using this example to consider how research partnerships operate in a way that is genuinely informed by ACCHO research principles.

### 4.1 ACCHO Research Principles Were Enacted at the Cultural Interface

It should be acknowledged that ACCHO research principles, and the ethical guidelines on which they were based, were designed to be applied by Aboriginal and Torres Strait Islander and non‐Indigenous researchers. Here participants and researchers are working at the ‘cultural interface’, where the aim is for Indigenous researchers and participants to maintain cultural authority within a Western structure [30]. Indigenous cultural authority in research is the principle that Indigenous communities hold the sovereign right to govern, interpret and determine the use of their own knowledge, cultural heritage, and data; shifting the research paradigm from studying *about* Indigenous peoples to Indigenous‐led research [1, 31]. Working at the cultural interface can cause tension and impact Indigenous researchers and participants asserting their cultural authority. Recognising the potential for this to occur in the photovoice study due to the different world views of Indigenous researchers and participants and non‐Indigenous researchers, a co‐design process was embedded in the methodology and all planned research activities were reviewed and approved as directed by the Apunipima and Congress Boards of Directors. Ensuring regular progress reviews with the project team where there were opportunities for frank discussions and openness to adaptation and change due to different ways of ‘knowing, being and doing’ [32] was key to respecting cultural authority. Indigenous research governance within the project, ‘the right to lead and/or participate at all stages of the research process and make decisions about the research process’ [33], resulted in changes to the research methods in response to participants identifying a more appropriate way of conducting workshops and gathering data. Here, the design of the study was first informed by the photovoice methodology, adapted by the working group with knowledge of working in communities, and then further adapted on engagement with participants who provided direction on workshop design. The researchers changed workshop facilitators and changed the format from group to individual workshops. The result was that participants felt comfortable and found it was convenient to participate individually and to engage in deep discussion with the researcher they were most familiar with. Deep insights into the cultural context of the images was made possible by participants storytelling where they were able to assert cultural authority by determining the use of their own knowledge, cultural heritage, and data. Within the context of Indigenous Lore, the vast body of cultural knowledge, customs, spiritual beliefs held by participants informed how the findings from the study would be represented in adding to their body of cultural knowledge. An agreed and upheld two‐way learning and sharing approach was crucial in non‐Indigenous researchers openness in deferring to Indigenous researchers' and participants' cultural authority in decision making, such as demonstrated in *the (iii) respect and relationships* theme, which described decisions about project managers leading questions in the workshops in response to participant preferences.

### 4.2 ACCHO Research Principles Were Supported by the Research

A *Murru Minya* study involved community representatives from approximately one‐third of ACCHOs in Australia. The ethical principles considered in this research to be most essential to research practice to these ACCHO representatives were sharing results with communities, research translation into policy and practice, and employment opportunities for Aboriginal and Torres Strait Islander peoples [9]. All are reflected in the Apunipima and Congress research principles, although as described, there were systemic constraints making ongoing employment difficult. Through the next steps in the broader project, which had funding for research translation, the findings from the photovoice study contributed to policy consultations. However, the immediate and tangible benefits to the participants of this study need to be examined. We found through evaluation that the photovoice participants enjoyed being part of the study, and there were short term benefits such as compensation for time, sharing stories and hearing other community members stories, however the long‐term benefits of research translation may be difficult to ascertain. For example, findings from this research contributed to (a) community workshops to develop community‐led solutions to improve food security, and (b) consultation on development of the National Strategy for Remote Aboriginal and Torres Strait Islander Food Security 2025–2035 (National Strategy). The National Strategy is still in the process of designing plans for implementation: meaning the outcomes are either unquantifiable or very long‐term, given this research took place in 2021 and the translation efforts are ongoing. We note that policy change is a slow, resource‐intensive and complex process, and such transformative impacts may be hard to document within the timeframes of academic publishing [34, 35].

### 4.3 Recommendations for Ethical Practice in Future Research

Research has found that Aboriginal and Torres Strait Islander people are more likely than non‐Indigenous people to perceive that researchers do not always engage in ethical research practice [8, 36]. During the course of the photovoice study, this team worked within structures that promoted engagement in an ongoing reflexive process with ACCHO research principles. The ACCHO research principles relevant to this study are based on the National Health and Medical Research Council (NHMRC) ethical guidelines for research conduct with Indigenous peoples. The two ACCHOs in this study applied a practical community lens to applying ethical guidelines as demonstrated in the NHMRC documents. In order to critically analyse how effectively the ACCHO ethical principles were operationalised in practice we used the CONSIDER statement [37] as a framework. We commend the journals that are now making the use of such tools compulsory in publishing research conducted with Aboriginal and Torres Strait Islander peoples [38, 39]. However, our main recommendation is the need for researchers to purposely plan and apply a strategy for applying and monitoring ethical guidelines at all levels and stages of the research as a priority at the conceptual stages of the research design.

This research also describes the importance of engaging with, and reporting on, intellectual and cultural property rights; relevant to multiple principles including *a strengths based approach which upholds culture*, and *respect and relationships*. Intellectual property rights need to be a part of agreements with Aboriginal organisations from the outset. A recent systematic review from this same authorship team found these considerations were rarely reported in participatory research to improve food security with Indigenous peoples [40]. In the photovoice study, the processes developed for consent and attribution meant only photographs that were approved by participants were shared, according to participant preference for attribution. This aligned with several of the ACCHO research principles. Whilst developing such processes might seem obvious for a method such as photovoice, where participants produce tangible materials that are clearly their own intellectual and/or cultural property, these principles apply to other types of research as well: the Aboriginal and Torres Strait Islander Quality Appraisal Tool notes cultural and intellectual property is both ‘tangible and intangible’ and includes, for example, stories [41].

### 4.4 Challenges in Applying ACCHO Research Principles

The photovoice study faced some challenges in applying the principles. The challenges of working in the cultural interface and strategies to manage this have been mentioned. However, it is working in an unknown community context that presents another challenge which required a willingness to be flexible, catering to the context and needs of community. Two opportunities to better address the principles were identified in the capacity building/strengthening principle. To further build community researcher engagement, the time between phases of the study meant continual communication was needed between phases, and the additional recruitment of community researchers for the photovoice study needed to start earlier. On reflection, providing more preliminary information to participants about options for community workshops and what these entailed may have better enabled participants to make more informed decisions. Further engagement with Community Researchers may have meant a local person/people facilitating the workshops and/or engaging in data analysis. This would have given the research more credence to operating at the cultural interface, further privileging Indigenous cultural authority [30].

A limitation of this article is that the process of reflection includes documentation from the perspective of individual researchers (including ACCHO staff) and does not consider reflections from the ACCHOs more broadly, or community or participant stakeholders. Photovoice participants, Community Researchers and members of the Community Advisory Group would have added a valuable and nuanced view of the implementation of the ACCHO research principles. The data included in this research does include an evaluation by photovoice participants, but it was limited in scope and did not explore the ACCHO research principles. Although there were three Aboriginal researchers co‐facilitating the photovoice study and co‐authoring this paper, one of whom has previously lived in one of the participating communities, it is a limitation not to have included current community members' perspectives on the implementation of ACCHO research principles. We therefore acknowledge the presence of confirmation bias that this paper comprises Indigenous and non‐Indigenous researchers reflecting on their own research.

## 5 Conclusion

This article has shared the outcomes of an ACCHO research principles synthesis and research reflection to determine how the research principles of two ACCHOs were applied in a photovoice study within a broader project where the ACCHOs were research partners. Our photovoice study processes demonstrated alignment with the ACCHO research principles. To improve capacity building, future research design should consider, for example, staffing capacity and project timelines, to further and better engage with Aboriginal and Torres Strait Islander peoples at the cultural interface. Researchers should consider intellectual and cultural property rights in planning, implementing and reporting research with Aboriginal and Torres Strait Islander peoples, and timeframes for research benefit to participants. National ethical guidelines [1, 2, 3] are reflected in ACCHO research principles and also tools such as the Research for Impact Tool [42], the QAT [43] and the CONSIDER statement [37], which should be used and reflected on to guide ethical research.

It is intended that the learnings from the process will inform our future research and contribute to the evidence in this field.

## Funding

This project is funded by a NHMRC Targeted Research grant (1179848). The contents of the published material are solely the responsibility of the Administering Institution, a Participating Institution or individual authors and do not reflect the views of NHMRC. E. Chappell was supported by a University of Queensland Research Training Program (RTP) Scholarship and a King & Amy O'Malley Postgraduate Scholarship. J.B. is supported by an NHMRC Investigator Grant (2017170).

## Ethics Statement

Research approval for the broader project was granted by the Chief Executive Officer of Apunipima Cape York Health Council, the Central Australian Aboriginal Congress Board, and ethical approval from the University of Queensland (2020/HE000636) and the Central Australian Human Research Ethics Committees (CA‐203701).

## Conflicts of Interest

The authors declare no conflicts of interest.

## Supporting information


**Data S1:** CONSIDER statement.
**Data S2:** ACCHO principles synthesis.
**Data S3:** Mapping of photovoice data to ACCHO research principles.

## Data Availability

The data that support the findings of this study are available from the corresponding author upon reasonable request.

## References

[1] Australian Institute of Aboriginal and Torres Strait Islander Studies , “AIATSIS Code of Ethics for Aboriginal and Torres Strait Islander Research,” 2020.

[2] National Health and Medical Research Council , Keeping Research on Track II: A Companion Document to Ethical Conduct in Research With Aboriginal and Torres Strait Islander Peoples and Communities: Guidelines for Researchers and Stakeholders (Commonwealth of Australia, 2018).

[3] National Health and Medical Research Council , Ethical Conduct in Research With Aboriginal and Torres Strait Islander Peoples and Communities: Guidelines for Researchers and Stakeholders (National Health and Medical Research Council, 2018).

[4] Lowitja Institute , M. Kennedy , and J. Bryant , “Ethics in Aboriginal and Torres Strait Islander Health Research: Discussion Paper,” 2024.

[5] United Nations General Assembly , “United Nations Declaration on the Rights of Indigenous Peoples,” 2007, https://www.un.org/development/desa/indigenouspeoples/wp‐content/uploads/sites/19/2018/11/UNDRIP_E_web.pdf.

[6] F. Collis and M. Kennedy , “Murru Minya: A National Exploration of Ethical Research and Research Ethics in Aboriginal and Torres Strait Islander Health and Medical Research,” Medical Journal of Australia 222, no. S2 (2025): S3–S5, 10.5694/mja2.52573. 39893584

[7] R. McGuffog , C. Chamberlain , J. Hughes , et al., “Murru Minya–Informing the Development of Practical Recommendations to Support Ethical Conduct in Aboriginal and Torres Strait Islander Health Research: A Protocol for a National Mixed‐Methods Study,” BMJ Open 13, no. 2 (2023): e067054, 10.1136/bmjopen-2022-067054. PMC9923310 36764710

[8] M. Kennedy , K. Booth , J. Bryant , et al., “How Well Are Researchers Applying Ethical Principles and Practices in Aboriginal and Torres Strait Islander Health and Medical Research? A Cross‐Sectional Study,” Medical Journal of Australia 222, no. S2 (2025): S49–S56, 10.5694/mja2.52572. 39893589

[9] F. Collis , K. Booth , J. Bryant , et al., “Aboriginal and Torres Strait Islander Community Experiences and Recommendations for Health and Medical Research: A Mixed Methods Study,” Medical Journal of Australia 222 (2025): S6–S15. 10.5694/mja2.52571 39893581

[10] L. J. Burchill , A. Kotevski , D. L. M. Duke , et al., “Ethics Guidelines Use and Indigenous Governance and Participation in Aboriginal and Torres Strait Islander Health Research: A National Survey,” Medical Journal of Australia 218, no. 2 (2023): 89–93, 10.5694/mja2.51757. PMC10952733 36253955

[11] N. J. Turner and K. L. Turner , “'Where Our Women Used to Get the Food': Cumulative Effects and Loss of Ethnobotanical Knowledge and Practice Case Study From Coastal British Columbia,” Botany 86, no. 2 (2008): 103–115, 10.1139/B07-020.

[12] National Aboriginal Community Controlled Health Organisation , “Aboriginal Community Controlled Health Organisations (ACCHOs),” 2022, accessed October 13, 2023, https://www.naccho.org.au/acchos/.

[13] O. Pearson , K. Schwartzkopff , A. Dawson , et al., “Aboriginal Community Controlled Health Organisations Address Health Equity Through Action on the Social Determinants of Health of Aboriginal and Torres Strait Islander Peoples in Australia,” BMC Public Health 20, no. 1 (2020): 1859. 10.1186/s12889-020-09943-4 PMC7716440 33276747

[14] M. Ferguson , E. Tonkin , J. Brimblecombe , et al., “Communities Setting the Direction for Their Right to Nutritious, Affordable Food: Co‐Design of the Remote Food Security Project in Australian Indigenous Communities,” International Journal of Environmental Research and Public Health 20, no. 4 (2023): 2936. 10.3390/ijerph20042936 PMC9957436 36833632

[15] Australian Bureau of Statistics , “National Aboriginal and Torres Strait Islander Health Survey,” 2024, accessed 20 August, 2025, https://www.abs.gov.au/statistics/people/aboriginal‐and‐torres‐strait‐islander‐peoples/national‐aboriginal‐and‐torres‐strait‐islander‐health‐survey/latest‐release.

[16] Apunipima Cape York Health Council , “Researching With Apunipima,” 2020, https://www.apunipima.org.au/wp‐content/uploads/2020/01/research_web.pdf.

[17] Central Australian Aboriginal Congress Aboriginal Corporation , “About Us,” 2022, accessed May 8, 2025, https://www.caac.org.au/about‐us/.

[18] Apunipima Cape York Health Council , “Position Statement: Food Security for Cape York,” 2020, https://www.apunipima.org.au/wp‐content/uploads/2020/02/positionstatement_foodsecuritycapeyork_final.pdf.

[19] Central Australian Aboriginal Congress Aboriginal Corporation , “Congress Research Strategy 2019–2023,” 2019, accessed October 12, 2023, https://www.caac.org.au/wp‐content/uploads/old‐files/Congress‐Research‐Strategy‐2019‐2023.pdf.

[20] Central Australian Aboriginal Congress Aboriginal Corporation , “Position Statement – Food Security,” 2022, https://www.caac.org.au/wp‐content/uploads/2022/05/Congress‐Food‐Security‐Position‐Statement.pdf.

[21] Central Australian Aboriginal Congress , “A Guide for Health Researchers Working With Aboriginal People in Central Australia,” 2021, https://www.caac.org.au/wp‐content/uploads/2022/05/Doing‐It‐Right‐web‐compressed.pdf.

[22] E. Chappell , E. Chan , C. Deen , et al., “Using Photovoice to Generate Solutions to Improve Food Security Among Families Living in Remote Aboriginal and/or Torres Strait Islander Communities in Australia,” BMC Public Health 24, no. 1 (2024): 785, 10.1186/s12889-024-18200-x. PMC10935805 38481178

[23] C. Wang and M. A. Burris , “Photovoice: Concept, Methodology, and Use for Participatory Needs Assessment,” Health Education & Behavior 24, no. 3 (1997): 369–387, 10.1177/109019819702400309. 9158980

[24] K. C. Hergenrather , S. D. Rhodes , C. A. Cowan , G. Bardhoshi , and S. Pula , “Photovoice as Community‐Based Participatory Research: A Qualitative Review,” American Journal of Health Behavior 33, no. 6 (2009): 686–698, 10.5993/Ajhb.33.6.6. 19320617

[25] C. Wang and M. A. Burris , “Empowerment Through Photo Novella: Portraits of Participation,” Health Education Quarterly 21, no. 2 (1994): 171–186, 10.1177/109019819402100204. 8021146

[26] Australian Human Rights Commission , “Self‐Determination,” n.d., accessed February 25, 2025, https://humanrights.gov.au/our‐work/aboriginal‐and‐torres‐strait‐islander‐social‐justice/self‐determination.

[27] Oxfam Australia , “Social Justice,” n.d., accessed February 25, 2025, https://www.oxfam.org.au/what‐we‐do/social‐justice/.

[28] Menzies School of Health Research , “Developing a Good Food System in Your Community. Information Sheet 1: Good Food Systems Overview,” 2016, https://www.menzies.edu.au/icms_docs/252289_Information_sheet_1.pdf.

[29] Lowitja Institute , “Annual Report 2020,” 2020, https://www.lowitja.org.au/wp‐content/uploads/2023/12/LI_AnnualReport_2020_FINAL3_5.pdf.

[30] M. Nakata , “The Cultural Interface,” Australian Journal of Indigenous Education 36, no. S1 (2007): 7–14.

[31] P. Anderson , Z. M. Diamond , T. Pham , et al., “Indigenous Rights‐Based Approaches to Decolonising Research Methodologies in Settler Colonial Contexts,” Frontiers in Research Metrics and Analytics 10 (2025): 1553208. Accepted manuscript. Published online 20250709, 10.3389/frma.2025.1553208. PMC12287929 40709213

[32] K. Martin and B. Mirraboopa , “Ways of Knowing, Being and Doing: A Theoretical Framework and Methods for Indigenous and Indigenist Re‐Search,” Journal of Australian Studies 27, no. 76 (2003): 203–214.

[33] Lowitja Institute , “Indigenous Data Governance and Sovereignty,” 2021, https://lowitja.org.au/wp‐content/uploads/2023/10/328550_data‐governance‐and‐sovereignty.pdf.

[34] C. Catalani and M. Minkler , “Photovoice: A Review of the Literature in Health and Public Health,” Health Education & Behavior 37, no. 3 (2010): 424–451. Accepted manuscript. Published online 2009/10/03, 10.1177/1090198109342084. 19797541

[35] M. Knowles , J. Rabinowich , T. Gaines‐Turner , and M. Chilton , “Witnesses to Hunger: Methods for Photovoice and Participatory Action Research in Public Health,” Human Organization 74, no. 3 (2015): 255–265.

[36] K. Booth , J. Bryant , F. Collis , et al., “Researchers' Self‐Reported Adherence to Ethical Principles in Aboriginal and Torres Strait Islander Health and Medical Research and Views on Improving Conduct: A Mixed Methods Study,” Medical Journal of Australia 222, no. S2 (2025): S16–S24, 10.5694/mja2.52570. 39893575

[37] T. Huria , S. C. Palmer , S. Pitama , et al., “Consolidated Criteria for Strengthening Reporting of Health Research Involving Indigenous Peoples: The CONSIDER Statement,” BMC Medical Research Methodology 19, no. 1 (2019): 173, 10.1186/s12874-019-0815-8. PMC6688310 31399058

[38] R. Maddox , A. Drummond , M. Kennedy , et al., “Ethical Publishing in ‘Indigenous’ Contexts,” Tobacco Control 33 (2024): e240–e245, 10.1136/tc-2022-057702. PMC11671947 36781227

[39] First Nations Health and Wellbeing , “The Lowitja Journal. Guide for Authors,” 2024, accessed 3 April, 2025, https://www.sciencedirect.com/journal/first‐nations‐health‐and‐wellbeing‐the‐lowitja‐journal/publish/guide‐for‐authors.

[40] E. Chappell , E. Chan , Y. Cadet‐James , et al., “The Use of Participatory Research to Address Food Security Among Indigenous Peoples in Select High Income Settler‐Colonised Countries: A Systematic Review,” Global Food Security 49 (2026): 100926, 10.1016/j.gfs.2026.100926.

[41] S. Harfield , O. Pearson , K. Morey , et al., The Aboriginal and Torres Strait Islander Quality Appraisal Tool: Companion Document (South Australian Health and Medical Research Institute, 2018).

[42] K. Tsey , K. Lawson , I. Kinchin , et al., “Evaluating Research Impact: The Development of a Research for Impact Tool,” Frontiers in Public Health 4 (2016): 160, 10.3389/fpubh.2016.00160. PMC4996827 27610359

[43] S. Harfield , O. Pearson , K. Morey , et al., “Assessing the Quality of Health Research From an Indigenous Perspective: The Aboriginal and Torres Strait Islander Quality Appraisal Tool,” BMC Medical Research Methodology 20, no. 1 (2020): 79, 10.1186/s12874-020-00959-3. PMC7147059 32276606

