# Supplementary material for: Reflections on the Application of Aboriginal Community Controlled Health Organisations' Research Principles in a Photovoice Food Security Study
Source: Health Promot J Austr. 2026 Sep 24;37(4):e70248. doi: 10.1002/hpja.70248 (PMC13612175; doi:10.1002/hpja.70248)
Supplement: Supplementary file 1 [file HPJA-37-0-s001.docx]

## Supplementary file:

## S1. CONSIDER statement

| Governance | |  |
| --- | --- | --- |
|  | | *Specify how and where you’ve addressed this in the paper, or note ‘Not applicable’ and provide reasoning.* |
|  | | The study reported in this paper, the application of ACCHO research principles in a photovoice study, (herein ‘this study’) is nested within a broader project, Giving Aboriginal and Torres Strait Islander children the best start in life: improving healthy food affordability and food security in remote Australia (The Remote Food Security Project, herein, the broader project). Consideration of the CONSIDER statement therefore include the broader project, the photovoice study (herein, the photovoice study), which sought to explore solutions to improve food security among parents and carers of children under five in remote Aboriginal and Torres Strait Islander communities, and the synthesis of research values and priorities from two Aboriginal Community Controlled Health Organisations (ACCHOs) and reflection on their application in the photovoice study (as noted, ‘this study’). |
| 1. | Describe partnership agreements between the research institution and Indigenous-governing organisation for the research (e.g., informal agreements through to MOU (Memorandum of Understanding) or MOA (Memorandum of Agreement)). | Governance processes are referred to throughout the paper, including in the **introduction**, and in the **findings** in the overarching theme, theme ii) *Aboriginal and Torres Strait Islander leadership, control and direction*, and theme iv) *a strengths-based approach which upholds culture.* |
| 2. | Describe accountability and review mechanisms within the partnership agreement that addresses harm minimisation. | All parties agreed to carry out the project in an ethical, responsible, diligent and competent manner in line with ethics clearances necessary for the project. Research and ethics approval are outlined in the **ethics** section in the **methods** of the paper. Also see description of Community Advisory Groups advising on adhering to community protocols in theme i) *Addressing community-identified needs and priorities).*  Specific consent processes for photographs including development of primary consent forms and secondary consent forms is described in the **findings** in themes iii) *respect and relationships* and iv) *a strengths-based approach which upholds culture* and also in the **discussion**. |
| 3. | Specify how the research partnership agreement includes protection of Indigenous intellectual property and knowledge arising from the research, including financial and intellectual benefits generated (e.g., development of traditional medicines for commercial purposes or supporting the Indigenous community to develop commercialisation proposals generated from the research). | Description of agreements including considerations for intellectual property is described in the **introduction.** |
| Prioritisation | |  |
| 4. | Explain how the research aims emerged from priorities identified by Indigenous stakeholders, governing bodies, funders, non-government organisation(s), stakeholders, consumers and empirical evidence. | The broader project emerged from Apunipima and Congress position statements on food security seen as a priority for Aboriginal and Torres Strait Islanders in Cape York and Central Australia (described in the **introduction** and used as data in **findings** section, in theme i), *Addressing community-identified needs and priorities*.  Apunipima identified photovoice as an appropriate method, as described in the **introduction**.  The aim of this study was to explore ACCHO research principles in relation to research conduct of the photovoice study; the significance of this is described in the last paragraph of the **introduction**. |
| Relationships (Indigenous stakeholders/participants and research team) | |  |
| 5. | Specify measures that adhere and honour Indigenous ethical guidelines, processes and approvals for all relevant Indigenous stakeholders, recognising that multiple Indigenous partners may be involved, e.g., Indigenous ethics committee approval, regional/national ethics approval processes. | Research approval pathways and reflections on working ethically at the cultural interface are described in the **ethics** section of the **methods**, and also in theme ii) in the **findings** *Aboriginal and Torres Strait Islander leadership, control and direction*). |
| 6. | Report how Indigenous stakeholders were involved in the research processes (i.e., research design, funding, implementation, analysis, dissemination/recruitment). | Indigenous involvement in funding and design is covered in the findings, theme i) *addressing community-identified needs and priorities*.  Indigenous involvement in the research process, from funding and decision making to implementation and analysis, is described throughout the paper including in the author roles statement of the **methods**, and the **findings** theme ii) *Aboriginal and Torres Strait Islander leadership, control and direction*. |
| 7. | Describe the expertise of the research team in Indigenous health and research. | Cultural background and expertise of authors is detailed in the **methods -** **researcher positionality and roles**. |
| Methodologies | |  |
| 8. | Describe the methodological approach of the research including a rationale of methods used and implication for Indigenous stakeholders, e.g., privacy and confidentiality (individual and collective) | The rationale for the photovoice method being identified by Apunipima is included in the **introduction**.  The development of this approach of research reflection is described in the researcher roles section of the **methods** – describing the senior researcher’s involvement in developing ACCHO principles and designing this study.  Confidentiality implications for participants were addressed through the development of processes and forms to ensure copyright and attribution of participants was protected when sharing their photographs, and to ensure the photographs, detailed in the **methods ethics** section and the **findings** in themes iv) *A strengths-based approach which upholds culture,* and iii) *respect and relationships* and in the **discussion.** |
| 9. | Describe how the research methodology incorporated consideration of the physical, social, economic and cultural environment of the participants and prospective participants (e.g., impacts of colonisation, racism and social justice), as well as Indigenous worldviews. | Recognition of the food security issues which impact remote communities is described in the **introduction** and emphasis on incorporation of traditional knowledges through a focus on community strengths is described in the **findings** in theme iv) *a strengths-based approach which upholds culture*. Indigenous worldviews (cultural considerations) through author roles are described in the **methods**, and throughout the **findings** by using ACCHO research principles as a framework. In the photovoice study, consideration for social and cultural elements included being flexible with workshop structure, described in the **findings** in theme iii) *respect and relationships*. |
| Participation | |  |
| 10. | Specify how individual and collective consent was sought to conduct future analysis on collected samples and data (e.g., additional secondary analyses; third-parties accessing samples (genetic, tissue, blood) for further analyses). | Detail on data access is included in the **methods – ethics** section. |
| 11. | Described how the resource demands (current and future) placed on Indigenous participants and communities involved in the research were identified and agreed upon, including any resourcing for participation, knowledge and expertise. | Voluntary participation is described in the **methods - ethics** section.  Reimbursement for time is described in the **findings** section in theme i) *addressing community-identified needs and priorities*. Participants were also informed that they would retain Intellectual Property and Cultural Property rights (described in **findings** themes iii) *respect and relationships* and iv) *A strengths-based approach which upholds culture*) and in the **discussion**. |
| 12. | Specify how biological tissue and other samples including data were stored, explaining the processes of removal from traditional lands, if done, and of disposal. | Data storage is described in the **methods – ethics** section. |
| Capacity | |  |
| 13. | Explain how the research supported the development and maintenance of Indigenous research capacity (e.g., specific funding of Indigenous researchers). | Development and maintenance of research capacity through NHMRC funding is described in **researcher positionality and roles** in the **methods** section. |
| 14. | Discuss how the research team undertook professional development opportunities to develop the capacity to partner with Indigenous stakeholders. | ACCHO’s provided capacity building through staffing, resources and expertise, covered in the **researcher positionality and roles** in the **methods** section.  Governance processes involving Aboriginal and Torres Strait Islander peoples which developed capacity of everyone in the teams in decision making around research is described in the **introduction**, and in the **findings** in theme ii) *Aboriginal and Torres Strait Islander leadership, control and direction*). Researcher capacity building through informal training and professional development is covered in the **researcher positionality and roles** in the **methods** section. |
| Analysis and interpretation | |  |
| 15. | Specify how the research analysis and reporting supported critical inquiry and a strength-based approach that was inclusive of Indigenous values. | The photovoice study was itself strengths-based, as described in the **findings** in the theme ‘a strength based approach which upholds culture’. The **researcher positionality and roles** in the **methods** section describes how Aboriginal authors were involved in results synthesis and the **methods** section- **Mapping the photovoice study documents to synthesised principles** describes one Aboriginal author’s role in developing the field notes. This paper is a critical inquiry into how the photovoice study’s design and conduct was informed by the research principles established by Apunipima and Congress, as described in the **introduction**. The study is by its nature inclusive of Indigenous values through profiling ACCHO research principles throughout the **findings.** |
| Dissemination | |  |
| 16. | Describe the dissemination of the research findings to relevant Indigenous governing bodies and peoples. | Community Advisory Groups roles in findings checking and dissemination is described in the **findings** in theme i) *addressing community-identified needs and priorities.*  Research dissemination is described in the **findings** theme iv) *a strengths-based approach which upholds culture.* |
| 17. | Discuss the process for knowledge translation and implementation to support Indigenous advancement (e.g., research capacity, policy, investment). | Knowledge translation is described in the **findings** in theme v) *responsible research (benefits and sustainability)* and further discussed in the **discussion.** |

## S2. ACCHO principles synthesis

| Theme name | Apunipima research value/priority | Congress research core value | Principles synthesis annotations |
| --- | --- | --- | --- |
| Overarching theme: Social justice, fairness, human rights lens | • Acknowledges the history and experiences of Aboriginal and Torres Strait Islander people and applies a social justice and human rights lens to the research | JUSTICE and FAIRNESS: Research commits to the principles of justice and fairness for equitable access to services and opportunities. Aboriginal community control is central. | Both organisations focus on justice. Fairness and human rights lens’ are synchronous. |
| i) Addressing community-identified needs and priorities | • Discipline and/or population group specific research which addresses the priorities and needs of communities identified at national, state and local levels | COMMITMENT: Research is respectful of culture. Engage with the community and stakeholders so that research priorities respond to community needs and improve the economic, cultural and social determinants of health. | Both organisations note the need to address community-identified needs and priorities. Apunipima notes the levels (national, state and local) and Congress notes this will address economic, cultural and social determinants of health. |
| ii) Aboriginal and Torres Strait Islander leadership, control and direction | • Is Aboriginal and Torres Strait Islander led or directed and which privileges the voices of Aboriginal and Torres Strait Islander People and honours their culture, kinship and knowledge systems | JUSTICE and FAIRNESS: Research commits to the principles of justice and fairness for equitable access to services and opportunities. Aboriginal community control is central. | Both organisations emphasise Aboriginal and Torres Strait Islander control (Congress), leadership or direction (Apunipima). |
| iii) Respect and relationships | • Is Aboriginal and Torres Strait Islander led or directed and which privileges the voices of Aboriginal and Torres Strait Islander People and honours their culture, kinship and knowledge systems | RESPECT & RELATIONSHIPS: Respect of cultural protocols and community and governance processes. Respectful behaviour includes awareness of different views, experience, values and priorities. Relationships are built and strengthened on this respect, trust and understanding. | This value name comes from the Congress value, which gives a guide to conducting respectful relationships. Apunipima notes the importance of research honouring people’s culture, kinship and knowledge systems. Apunipima emphasises privileging Aboriginal and Torres Strait Islander voices. |
| iv) Strengths based; upholding culture | • Is meaningful, strengths based and which benefits Aboriginal and Torres Strait Islander people  • Acknowledges the history and experiences of Aboriginal and Torres Strait Islander people and applies a social justice and human rights lens to the research  • Is Aboriginal and Torres Strait Islander led or directed and which privileges the voices of Aboriginal and Torres Strait Islander People and honours their culture, kinship and knowledge systems  Research which incorporates a focus on culture as an enabler of wellbeing | UPHOLD CULTURE: Research upholds and supports culture. Cultural distinctiveness and the lived history in community is recognised. All research operates within a cultural safety framework.  COMMITMENT: Research is respectful of culture. Engage with the community and stakeholders so that research priorities respond to community needs and improve the economic, cultural and social determinants of health. | For Apunipima, a strengths-based approach is linked to benefits (covered in next value) and acknowledging history. Upholding culture comes from the Congress core value name and is reflected in both organisations’ values – with Apunipima noting the importance of ‘honour[ing] culture, kinship and knowledge systems’, linking culture to wellbeing and Congress mentions a cultural safety framework. |
| v) Responsible research (Benefits and sustainability) | • Is meaningful, strengths based and which benefits Aboriginal and Torres Strait Islander people  • Is sustainable, adds to an evidence base, and which facilitates knowledge translation to influence policy and practice to improve outcomes. | SHARING: Collaborative research so that learnings benefit the community, knowledge is shared, and research outcomes are translated into policy and practice.  RESPONSIBILITY: High quality, ethical, coordinated research is planned, approved, implemented and completed. Engagement through agreed ways of communicating leads to better policy, practice and service. A focus on sustainability means long lasting, meaningful outcomes, shared by all. | Both organisations emphasised the need for research to be meaningful and sustainable. Apunipima noted the importance of knowledge translation; Congress noted the importance of collaboration, communication, ethics, planning, approval, implementation and completion. |
| vi) Capacity building/strengthening | • Builds and/or strengthens the capacity and capability of Aboriginal and Torres Strait Islander people to lead and/or effectively engage in research and research related activities at community, state, national and international level  • Research which informs the establishment and maintenance of a qualified, productive and culturally competent workforce. | Implicit in core values, and explicit in ‘doing it right’ guide – Working with Aboriginal researchers, language speakers, community navigators and advisors. | Apunipima explicitly includes capacity building/strengthening in its research values, and priorities. Congress implies this through values (e.g. ‘collaborative research’ as part of the sharing value) but also includes explicit guidance for this in the ‘Doing it Right’ guide. |

**S3. Mapping of photovoice data to ACCHO research principles**

| Theme name | How the study or project addressed this theme (summary) |
| --- | --- |
| Overarching theme: Social justice, fairness, human rights lens | - Process consistent with human rights principles of participation and self-determination - Community voices heard fairly throughout the project, informing local solutions and government consultation   Representation was equitable through governance structure   - Knowledge sharing and reflection was equitable through identifying community priorities and solutions - Food security focuses on the human right to food, and social justice focuses on in the equal distribution of benefits in society |
| i) Addressing community-identified needs and priorities | - Apunipima and Congress identified priority of food security, and the priority population, in position statements and Congress’ research strategy - Consultation with and approval from remote boards (Congress) and Community Health Action Teams (Apunipima) - Community Advisory Groups in communities provided direction and guidance in each community ensuring community and cultural protocols were adhered to - Photovoice photography prompt asked about needs and priorities relating to food security - Social and cultural determinants of health addressed through adhering to cultural protocols, ensuring that participants understood that any information that related to sensitive cultural practices would remain the intellectual property of the participant/s and that participants would be in control of what was included in findings, reports, publications and presentation, being flexible with workshop structure to accommodate community needs and participants preferences - Community Advisory Groups were consulted on engagement and adherence to community protocols - Participants were engaged in decision making regarding workshops and processes and actively participated in workshops and taking photographs |
| ii) Aboriginal and Torres Strait Islander leadership, control and direction | - (leadership) Governance structure: Chief Investigators include four Aboriginal and Torres Strait Islander members.YCJ is also a supervisor for the PhD candidate (ELC). One of the Project Managers who led implementation is an Aboriginal woman (CD). There are Aboriginal and Torres Strait Islander members on working groups and the Community Advisory Groups - (control) Apunipima and Congress research approval pathways allowed for Aboriginal and Torres Strait Islander control of research - (direction) Participants checked themes and CD and ES contributed to thematic analysis, allowing for Aboriginal direction of research - The community-led framework meeting faciliated Aboriginal and Torres Strait Islander control of knowledge translation of suggested solutions and priorities |
| iii) Respect and relationships | - Existing relationships with participants were respected through strategies such as the recruitment strategy and adapting the protocol for Project Managers to lead the second workshop - Relationships were built with participants through providing transport and personal photographs, and developed over time through extensive community engagement - Community direction around competing priorities and sorry business were followed, leading to changes in schedule or workshop format (from group workshops to mostly individual workshops) - There were multiple opportunities for participants to consider if and how photographs and stories should be included in data set - Different views were handled with respect and consideration, privileging Indigenous voices about how they wanted to proceed |
| iv) Strengths based; upholding culture | - Governance structure included Aboriginal and Torres Strait Islander people at every level informing decision-making - Apunipima and Congress led implementation as partners who best know community as they provide services to the community - Findings informed community prioritisation workshops which informed community-led framework, which informed consultation on policy - Photography prompt and discussion questions provided the opportunity to explore community strengths - Consent forms and processes protected cultural and intellectual property, with the focus on consent for participation, for use of photos, for permission to use photos of others, and then going back to check on further consent for publication with photos of people - Informed by ACCHO research principles and participatory research methodology |
| v) Responsible research (Benefits and sustainability) | - Findings informed community prioritisation workshops which informed the community-led framework and then informed consultation regarding translation of findings into policy to improve food security outcomes - Community-led framework which provides opportunities for communities to have input from a local perspective - Evaluation data highlighted that the photovoice data was relevant and included solutions to improve food security, not previously identified in the broader project - Ethics approval process was collaborative and included planning, approval, implementation and completion - Two-way communication processes provided results back to participants and allowed for feedback |
| vi) Capacity building/strengthening | - Capacity building and strengthening occurred across the structures of the project; within Apunipima and Congress, with Indigenous and other researchers, Project Managers, community researchers, community stakeholders and advisory groups, and participants - Project Managers were supported to lead the second photovoice workshops - Community Researchers were employed to support recruitment and advise on implementation in three communities - Participants were supported (at their request) to take photographs, and build stories. Protocol and resources allowed for multiple ways to participate including using their own existing photos, providing and replacing lost/damaged cameras - Three participants continued engagement with the project by participating in community prioritisation workshops, contributing to community-developed priorities and solutions |
